# Gallic acid suppresses the progression of triple-negative breast cancer HCC1806 cells *via* modulating PI3K/AKT/EGFR and MAPK signaling pathways

**DOI:** 10.3389/fphar.2022.1049117

**Published:** 2022-11-29

**Authors:** Si Lin, Hui-Zhen Qin, Ze-Yu Li, Hua Zhu, Li Long, Li-Ba Xu

**Affiliations:** ^1^ Guangxi Key Laboratory of Zhuang and Yao Ethnic Medicine, Guangxi University of Chinese Medicine, Nanning, China; ^2^ Guangxi Scientific Research Center of Traditional Chinese Medicine, Guangxi University of Chinese Medicine, Nanning, China; ^3^ Guangxi International Zhuang Medicine Hospital, Guangxi University of Chinese Medicine, Nanning, China

**Keywords:** triple-negative breast cancer, gallic acid, proliferation, apoptosis, PI3K/AKT/EGFR signaling pathway, MAPK signaling pathway

## Abstract

Triple-negative breast cancer (TNBC) is a severe threat to women’s health because of its aggressive nature, early age of onset, and high recurrence rate. Therefore, in this study, we aimed to evaluate the anti-tumor effects of Gallic acid (GA) on the TNBC HCC1806 cells *in vitro*. The cell proliferation was detected by MTT and plate clone formation assays, cell apoptosis, cell cycle, and mitochondrial membrane potential (MMP) were analyzed by flow cytometry and Hoechst 33258 staining assays, and the intracellular reactive oxygen species (ROS) accumulation were also investigated. Real-Time PCR and western blot were examined to explore the mechanism of action. The results indicated that GA suppressed HCC1806 cells proliferation and promoted HCC1806 cells apoptosis. Meanwhile, GA treatment changed the morphology of the HCC1806 cells. In addition, GA blocked the HCC1806 cells cycle in the S phase, and it induced cells apoptosis accompanied by ROS accumulation and MMP depolarization. Real-Time PCR results suggested that GA increased Bax, Caspase-3, Caspase-9, P53, JINK and P38 mRNA expression, and decreased Bcl-2, PI3K, AKT and EGFR mRNA expression. Western blotting results suggested that GA increased Bax, cleaved-Caspase-3, cleaved-Caspase-9, P53, P-ERK1/2, P-JNK, P-P38 proteins expression, and decreased Bcl-2, P-PI3K, P-AKT, P-EGFR proteins expression. Furthermore, molecular docking suggested that GA has the high affinity for PI3K, AKT, EGFR, ERK1/2, JNK, and P38. In conclusion, GA could suppress HCC1806 cells proliferation and promote HCC1806 cells apoptosis through the mitochondrial apoptosis pathway and induces ROS generation which further inhibits PI3K/AKT/EGFR and activates MAPK signaling pathways. Our study will provide some new references for using GA in the treatment of TNBC.

## Introduction

Breast cancer (BC) is a malignant tumor that seriously threatens women’s life and health worldwide (Akram et al., 2017). According to the statistics, an estimated 2.3 million new cases of BC worldwide in 2020 (Sung et al., 2021). Triple-negative breast cancer (TNBC), which accounts for 20% of all BC, is a type of BC that is negative for estrogen receptor (ER), progesterone receptor (PR) and human epidermal growth factor receptor 2 (HER-2), resulting in a meager survival rate due to its aggressiveness, malignancy and poor prognosis (Oh et al., 2017; Hon et al., 2016). Due to its unique molecular phenotype, TNBC is insensitive to endocrine and molecular targeted therapy. Currently, the main treatment methods are local surgical excision and systemic chemotherapy. However, conventional postoperative adjuvant radiotherapy is less effective and has a higher metastasis and recurrence rate, so TNBC has became the most treatment-challenging BC subtype (Denkert et al., 2017). The doxorubicin, represented by adriamycin, is among the most effective drugs used in chemotherapy for TNBC. However, its usage is greatly limited due to its tendency to develop resistance to drugs. (Angelini et al., 2013; Woo et al., 2016; Aydinlik et al., 2017). Therefore, it is urgent to find a novel anti-tumor drug with effective, fewer side effects and less toxicity for the TNBC treatment.

As we all know, an increasing number of studies have demonstrated that plant extracts, especially traditional Chinese medicine (TCM) extracts have apoptosis-promoting effects on a wide range of cancer cells. For example, Chen et al. (2020) revealed that Ethanol Extract of *Brucea javanica* Seed could suppress TNBC cell proliferation and promote cell apoptosis. Li et al. (2020) found that Extracts of Cordyceps sinensis could inhibit BC growth by promoting M1 macrophage polarization *via* NF-*κ*B pathway activation. Therefore, the search for new anti-tumor drugs from plant extracts has attracted the attention of a wide range of researchers.

Gallic acid (GA) is a polyphenol compound with various biological activities such as antioxidant, anti-inflammatory, anti-tumor, anti-viral and anti-bacterial (Lin et al., 2020; Bai et al., 2021). In current times, numerous research studies have confirmed the anti-tumor effect of GA, and it is said to be a potential anti-cancer agent (Zhang N et al., 2019; Zeng et al., 2020; Lin Q et al., 2021; Jiang et al., 2022). At the same time, a growing number of studies reported that inhibition of tumor cell proliferation and induction of apoptosis are the keys to active anti-tumor drugs, and both radiotherapy and chemotherapy are closely related to the induction of apoptosis (Hickman, 1992; Gong et al., 2018; Jan and Chaudhry, 2019; Hu et al., 2021). Apoptosis pathways include the mitochondrial pathway, the death receptor and the endoplasmic reticulum pathway, and the mitochondrial pathway plays a crucial role. Some studies showed a strong link between mitochondrial morphology and cancer disease (Maycotte et al., 2017). B-cell lymphoma 2 (Bcl-2) and Bcl-2-associated X protein (Bax) play an important role in the regulation of the mitochondrial apoptosis pathway (Lv et al., 2020; Ma et al., 2020). In response to various apoptosis stimulus signals, Bax protein conformation changes and translocates to the outer mitochondrial membrane, forming a homodimer with a microporous structure, stimulating the release of pro-apoptotic factors such as cytochrome C into the cytoplasm, forming a multimeric complex with apoptotic protease-activating factor-1, promoting the self-activation of cysteine aspartate protease-9 (caspase-9) precursor, which activates caspase-3 and ultimately leads to apoptosis (Liu et al., 2020; Montero et al., 2020). In addition, several studies have reported that dysregulation of the Phosphoinositide-3-kinase (PI3K)/protein kinase B (AKT) signaling pathway is closely associated with the development of breast cancer (Abdullah et al., 2021; Guo et al., 2021). And activated AKT signaling pathway promotes breast cancer cell growth, survival and metastasis (Lin X et al., 2021; Ock and Kim, 2021; Shin et al., 2021). Epidermal growth factor receptor (EGFR) is a tyrosine kinase that promotes cell proliferation and inhibits apoptosis, and its high expression can promote neovascularization and induce tumorigenesis, invasion and metastasis (Ohmori et al., 2021; Lee et al., 2018). Mitogen-activated protein kinase (MAPK) signaling pathways pathway has three sub-pathways, including the ERK, JNK and p38 MAPK sub-pathways, with the ERK pathway reported to be essential for cell survival. At the same time, JNK and p38 MAPK were associated with apoptosis (Yue and Lopez, 2020). In a word, PI3K/AKT/EGFR and MAPK signaling pathways play a crucial role in cancer cell proliferation and tumor progression, and a previous study has confirmed that EGFR is a target for TNBC treatment (Liao et al., 2019).

Although some studies have confirmed the anti-tumor activity of GA against TNBC, for example, Lee et al. (2017) found that GA induced apoptosis in MDA-MB-231 cells through the P38MAPK/P27/P21 signaling axis, Khorsandi et al. (2020) found that GA induced ferroptosis in MDA-MB-231 cells through the production of ROS, and Moghtaderi et al. (2018) found that GA combined with curcumin induced apoptosis in MDA-MB-231 cells by decreasing the expression of Bcl-2 and increasing the expression of Bax, cleaved-caspase3 and PARP, the exact mechanism of GA action on HCC1806 cells is still unknown. Hence, the current study was conducted to investigate whether GA could inhibit HCC1806 cells progression, and our experiments evaluated the effects of GA on proliferation, apoptosis, cycle, and mitochondrial membrane potential of HCC1806 cells for the first time. In addition, we investigated whether GA could regulate the PI3K/AKT/EGFR and MAPK signaling pathways and thus promote HCC1806 cells apoptosis by promoting ROS generation, which would provide further references for the use of GA in the treatment of TNBC.

## Materials and methods

### Reagents

GA with 99% purity was purchased form Shanghai Macklin Biochemical Co., Ltd. (Shanghai, China). RPMI-1640 medium, crystal violet and Cell Cycle Assay Kit were purchased form Jiangsu KeyGEN BioTECH Corp., Ltd. (Nanjing, China). Fetal bovine serum (FBS) was purchased form Gibco (United States). MTT and Hoechst 33258 solution were acquired from Beijing Solarbio Science & Technology Co., Ltd. (Beijing, China). Annexin V-FITC Apoptosis Detection Kit was acquired from BD Biosciences (Becton Dickinson, United States). Mitochondria Staining Kit (JC-1) was acquired from MultiSciences (Lianke) Biotech Co., Ltd. (Hangzhou, China). ROS Assay Kit was acquired from Beyotime Institute of Biotechnology (Shanghai, China). The antibodies against Caspase-3, cleaved-Caspase-3, cleaved-Caspase-9, Bax, Bcl-2, PI3K, P-PI3K, AKT, P-AKT, JNK, P-JNK, ERK1/2, P-ERK1/2, P38, P-P38, EGFR, P-EGFR, GAPDH were obtained from Cell Signaling Technology (CST, United States). The antibodies against Caspase-9 and P53 were obtained from Proteintech Group, Inc. (Wuhan, China).

### Cell culture and drug preparation

The human TNBC HCC1806 and MDA-MB-468 cells line were purchased form Jiangsu KeyGEN BioTECH Corp., Ltd. (Nanjing, China). HCC1806 cells were cultured with RPMI-1640 medium supplemented with 10% FBS and 1% penicillin/streptomycin at 37°C in a humidified incubator with 5% CO_2_. GA was prepared into a storage solution (1,000 μM/L) with FBS-free RPMI-1640 medium, then stored at -20°C. Dissolve the GA stock solution and dilute it to the appropriate concentration with FBS-free RPMI-1640 medium before using it.

### Cell viability detection

HCC1806 cells were (5 × 10^3^ cells/well) seeded into 96-well plates overnight and then treated with GA (0, 200, 250, and 300 μM) for 24, 48, and 72 h. The MTT (5 mg/ml) reagent was added to each well (20 μL) and cultured for another 4 h. Next, dimethyl sulfoxide (DMSO) was added to each well (150 μL), and the absorbance was recorded at 490 nm by a Enzyme labeling instrument (BioTek, United States). MDA-MB-468 cells were (5 × 10^3^ cells/well) seeded into 96-well plates overnight and then treated with GA (0, 3, 6, 12, 24, 48, 96, 192, 288, and 384 μM) for 24 h. Other steps are the same as those of HCC1806 cells (Yu et al., 2016).

### Cellular morphology analysis

HCC1806 cells were plated in 6-well plate for 24 h, and then treated with GA (0, 200, 250, and 300 μM) for 24 h. The cell morphology was observed by an inverted microscope (Olympus, Japan) (Zeng et al., 2020).

### Plate clone formation assay

HCC1806 cells were (1 × 10^3^ cells/well) seeded into 6-well plates overnight, and then treated with GA (0, 200, 250, and 300 μM) for 24 h. Subsequently, removed the media and added to fresh media, and allowed to grow undisturbed for 10 days. Cells were fixed with 4% paraformaldehyde for 15 min and stained with crystal violet solution for 15 min. The plates were dried and photographed, and ImageJ Software counted the number of colonies (Zeng et al., 2020).

### Cell cycle analysis

HCC1806 cells were seeded into 6-well plates overnight, and then treated with GA (0, 200, 250, and 300 μM) for 24 h. Subsequently, cells were digested and collected, washed once in PBS, fixed for 3 h at 4°C in 2 ml of 75% ethanol, then washed once in PBS, 500 μL PI/RNase dye solution was added and incubated for 20 min at room temperature and protected from light, and the fluorescence intensity of HCC1806 cells were detected by flow cytometry (Dakewe Biotech Co., Ltd., Shenzhen, China). (Zeng et al., 2020).

### Hoechst 33258 staining

HCC1806 cells were seeded into 6-well plates overnight, and then treated with GA (0, 200, 250, and 300 μM) for 24 h. Next, cells were fixed with 4% paraformaldehyde for 15 min, followed by incubation with Hoechst 33258 solution for 10 min. Cells were observed and photographed under an inverted fluorescence microscope (Nikon, Japan) (Zeng et al., 2020).

### Cell apoptosis assay

HCC1806 cells were seeded into 6-well plates overnight, and then treated with GA (0, 200, 250, and 300 μM) for 24 h. Subsequently, cells were digested and collected, then washed with cold PBS. Moreover, add 500 μL of Binding Buffer to each tube, resuspension by centrifugation, add 5 μL of Annexin V-FITC and 10 μL of PI staining solution to each tube, and incubate for 15 min at 25°C, protected from light, and the apoptosis rate of HCC1806 cells were detected by flow cytometry (Dakewe Biotech Co., Ltd., Shenzhen, China). (Zeng et al., 2020).

### MMP level assessment

HCC1806 cells were seeded into 6-well plates overnight, and then treated with GA (0, 200, 250, and 300 μM) for 24 h. Subsequently, cells were digested and collected, and washed with PBS. Then add 1 ml JC-1 Staining Buffer to each tube and incubated for 30 min at room temperature and protected from light, and the depolarization rate of HCC1806 cells were detected by flow cytometry (Dakewe Biotech Co., Ltd., Shenzhen, China) (Zeng et al., 2020).

### Intracellular ROS production

HCC1806 cells were seeded into 6-well plates overnight, and then treated with GA (0, 200, 250, and 300 μM) for 24 h. Subsequently, 1 ml DCFH-DA working solution was added to each well and incubation at 37°C for 30 min. Finally, cells were observed and photographed under an inverted fluorescence microscope (Nikon, Japan), and fluorescence intensity was detected by ImageJ Software (Zeng et al., 2020).

### Quantitative Real-Time polymerase chain reaction (QRT-PCR) analysis

HCC1806 cells were seeded into 6-well plates overnight, and then treated with GA (0, 200, 250, and 300 μM) for 24 h. Cells were processed and harvested according to the manufacturer’s instructions, total RNA was extracted using a TRIzol reagent kit (Thermo Fisher Scientific, United States). RNA was reverse-transcribed with cDNA reverse transcription kits (Thermo Fisher Scientific, United States). The sequences of PCR primers were design and synthesis form Sangon Biotech (Shanghai, China) Co., Ltd. (Shanghai, China). And the sequences as followed in Table 1. The relative mRNA expression levels were analyzed by 2^−ΔΔCT^ method and normalized to GAPDH (Zeng et al., 2020).

**TABLE 1:** Sequences of real-time polymerase chain reaction (RT-PCR) primer.

	Forward primer 5′ to 3′	Reverse primer 5′ to 3′
Bax	AGC​GAC​TGA​TGT​CCC​TGT​CTC​C	AGA​TGG​TGA​GTG​AGG​CGG​TGA​G
Bcl-2	TCG​CCC​TGT​GGA​TGA​CTG​AGT​AC	ACA​GCC​AGG​AGA​AAT​CAA​ACA​GAG​G
Caspase-3	GTG​GAG​GCC​GAC​TTC​TTG​TAT​GC	TGG​CAC​AAA​GCG​ACT​GGA​TGA​AC
Caspase-9	GAC​CAG​AGA​TTC​GCA​AAC​CAG​AGG	AAG​AGC​ACC​GAC​ATC​ACC​AAA​TCC
P53	GCC​CAT​CCT​CAC​CAT​CAT​CAC​AC	GCA​CAA​ACA​CGC​ACC​TCA​AAG​C
PI3K	GGA​AGC​AGC​AAC​CGA​AAC​AAA​GC	TCC​ACC​ACT​ACA​GAG​CAG​GCA​TAG
AKT	AGA​TGC​AGC​CAC​CAT​GAA​GAC​ATT​C	ACC​AGT​CTA​CTG​CTC​GGC​CAT​AG
EGFR	TAC​TTG​GAG​GAC​CGT​CGC​TTG​G	CTC​TTC​CGC​ACC​CAG​CAG​TTT​G
ERK1/2	TCG​CCG​AAG​CAC​CAT​TCA​AGT​TC	TCC​TGG​CTG​GAA​TCT​AGC​AGT​CTC
JNK	ACT​ACA​GAG​CAC​CCG​AGG​TCA​TC	TTT​CTC​CCA​TGA​TGC​ACC​CAA​CTG
P38	GCA​GAG​CGA​TGA​GGC​CAA​GAA​C	GCG​TCC​AGC​ACC​AGC​ATC​TTC
GAPDH	CAG​GAG​GCA​TTG​CTG​ATG​AT	GAAGGCTGGGGCTCATTT

### Western blotting

HCC1806 cells were seeded into 6-well plates overnight, and then treated with GA (0, 200, 250, 300 μM) for 24 h. After that, The culture solution was removed and the 6-well plates were transferred to ice and washed 3 times with PBS. Then 300 μL RIPA lysis buffer solution was added to each well, lysed on ice for 30 min and then transferred to EP tubes and centrifuged. Protein concentrations were detected by the Enhanced BCA Protein Assay Kit (Beyotime Institute of Biotechnology, Shanghai, China). The protein samples were separated by 10% or 8% SDS-PAGE and then transferred to a polyvinylidene difluoride (PVDF) membrane (Millipore, United States). BSA (5%) (Beijing Solarbio Science & Technology Co., Ltd., Beijing, China) was used to block the membrane for 1 h, and then the primary antibodies Caspase-3 (1:1000), cleaved-Caspase-3 (1:500), cleaved-Caspase-9 (1:1000), Bax (1:1000), Bcl-2 (1:500), PI3K (1:1000), P-PI3K (1:1000), AKT (1:1000), P-AKT (1:1000), JNK (1:1000), P-JNK (1:500), ERK1/2 (1:1000), P-ERK1/2 (1:1000), P38 (1:1000), P-P38 (1:1000), EGFR (1:1000), P-EGFR (1:500), P53 (1:1000), Caspase-9 (1:1000) and GAPDH (1:1000) were incubated overnight at 4°C, followed by incubation with the secondary antibodies (1:3000, Proteintech Group, Inc., Wuhan, China) at room temperature for 1 h. Protein bands were visualized using the ECL Plus western blotting detection reagents (Shanghai Epizyme Biomedical Technology Co., Ltd., Shanghai, China) following the manufacturer’s instructions. The relative expression of proteins were analyzed by ImageJ Software (Zeng et al., 2020).

### Molecular docking

To further explore the molecular target of GA in TNBC cells, The molecular docking of GA with PI3K, ERK1/2, JNK, EGFR, AKT and P38 were carried out by autodock 4.2.6 software. It is generally believed that the binding energy <0 indicates that the small molecule components of the ligand and the target protein of the receptor can bind independently. The lower the binding energy, the more stable the molecular conformation, the greater the possibility of action, and the greater the absolute value of the binding energy, the better the binding between the ligand and the receptor.

### Statistical analysis

The results were shown as the mean *±* standard deviation (SD). Each experiment was repeated at least six times. All the data of this paper were analyzed with SPSS 22.0 (IBM, United States). Comparison between groups using one-way ANOVA followed by Tukey’s test. *p* < 0.05 was defined as statistically significant.

## Results

### GA inhibits HCC1806 cells viability

**FIGURE 1:**
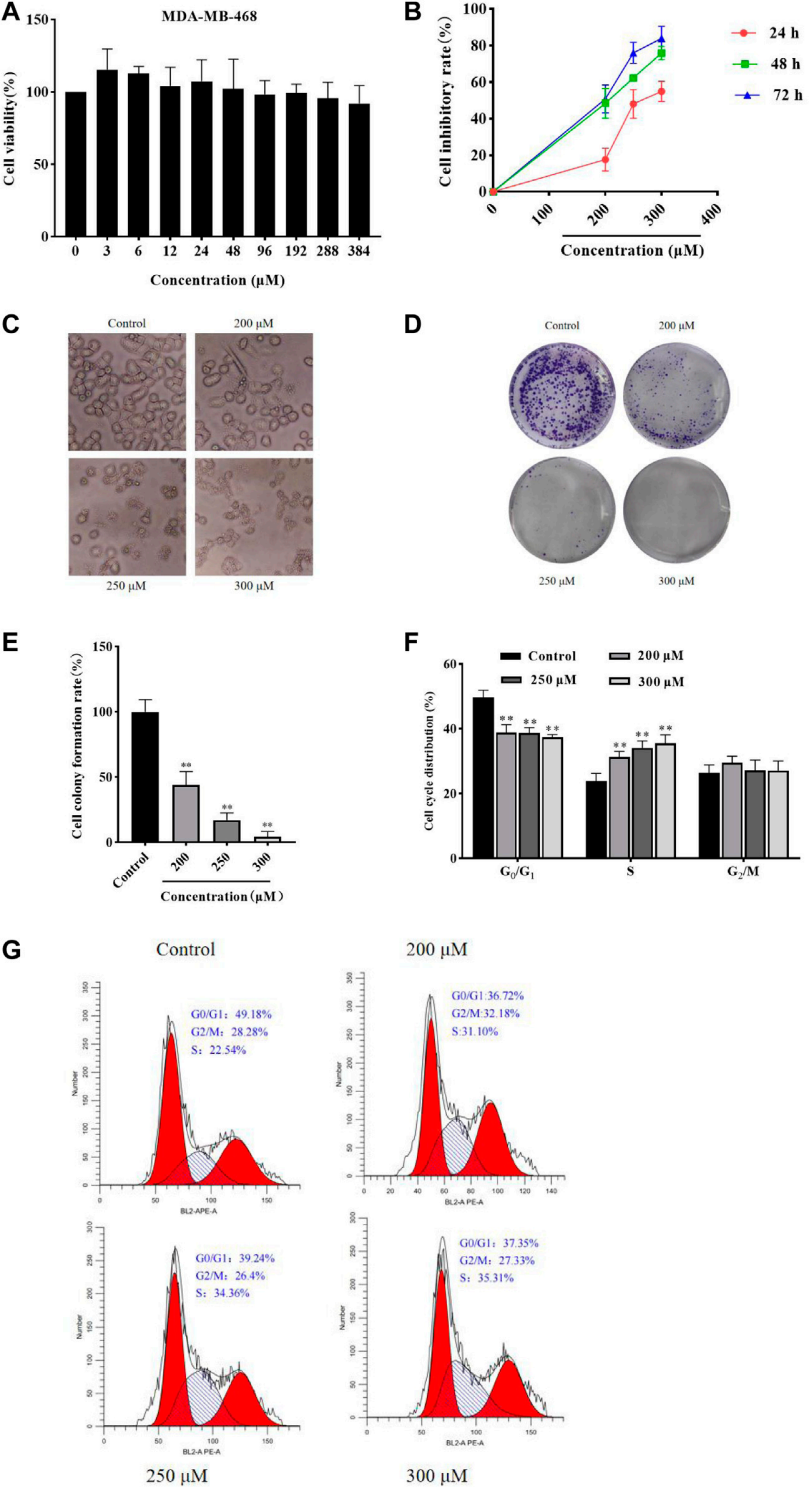
GA inhibited HCC1806 cells viability and affected cells morphology, and affected HCC1806 cells proliferation and cycle distribution. **(A)** The MDA-MB-468 cells viability was detected by MTT assay. **(B)**The HCC1806 cell inhibitory rate was detected by MTT assay. **(C)** The morphology changes of HCC1806 cells treated with different concentrations of GA were observed under an inverted microscope. **(D,E)** Effect of GA on the colony formation rate of HCC1806 cells. **(F,G)** The cell cycle distribution of HCC1806 were assessed by flow cytometry. Date were expressed as means ± SD. Compared with the control group, ^**^ *p* < 0.01.

MTT assay was performed to detect the MDA-MB-468 and HCC1806 cells viability. As shown in Figure 1A, GA has no obvious inhibitory effect in MDA-MB-468 cells after GA treatment for 24 h. While GA has notable inhibitory effect in HCC1806 cells. As shown in Figure 1B, compared with the control group, GA treatment significantly increased the cell inhibitory rate. These results suggested that GA notably inhibited HCC1806 cell viability.

### GA changes HCC1806 cells morphology

After different concentrations of GA (0, 200, 250, and 300 μM) treatment HCC1806 cells for 24 h, the changes in cell morphology could be observed under an inverted microscope. As shown in Figure 1C, the control group cells still adhered to the wall, with clear cell contour, high cell density and in a great condition. However, the cells in GA treatment groups were shrank, the nucleus was fragmented, the cell density decreased and cells were in a state of apoptosis.

### GA increases HCC1806 cells cytotoxic activity

The plate clone formation assay was performed to assess the effect of GA on HCC1806 cells cytotoxic activity. As shown in Figures 1D,E, compared with the control group, GA treatment was significantly decreased the number of colonies of HCC1806 cells, and the number of colonies decreased with increasing GA concentration. These results suggested that GA notably enhanced HCC1806 cells cytotoxic activity.

### GA blocked HCC1806 cells cycle in S phase

The flow cytometry was performed to assess the effects of GA on HCC1806 cells cycle. As shown in Figures 1F,G, in the control group, the percentage of HCC1806 cells in G_0_/G_1_ phase was 50.47% ± 1.63%, in S phase was 23.32% ± 2.25%, and in G_2_/M phase was 25.88% ± 2.13%. Compared with the control group, the percentage of G_0_/G_1_ phase in GA treatment groups (200, 250, 300 μM) were 39.18% ± 0.30%, 37.27% ± 0.22%, 36.87% ± 0.14%, respectively, the percentage of G_0_/G_1_ phase in GA treatment groups were significantly decreased. Compared with the control group, the percentage of S phase in GA treatment groups (200, 250, 300 μM) were 31.37% ± 1.07%, 34.06% ± 1.03%, 34.16% ± 1.27%, respectively, the percentage of S phase in GA treatment groups were significantly increased. These results suggested that GA blocked HCC1806 cells cycle in S phase.

### GA induces HCC1806 cells apoptosis

**FIGURE 2:**
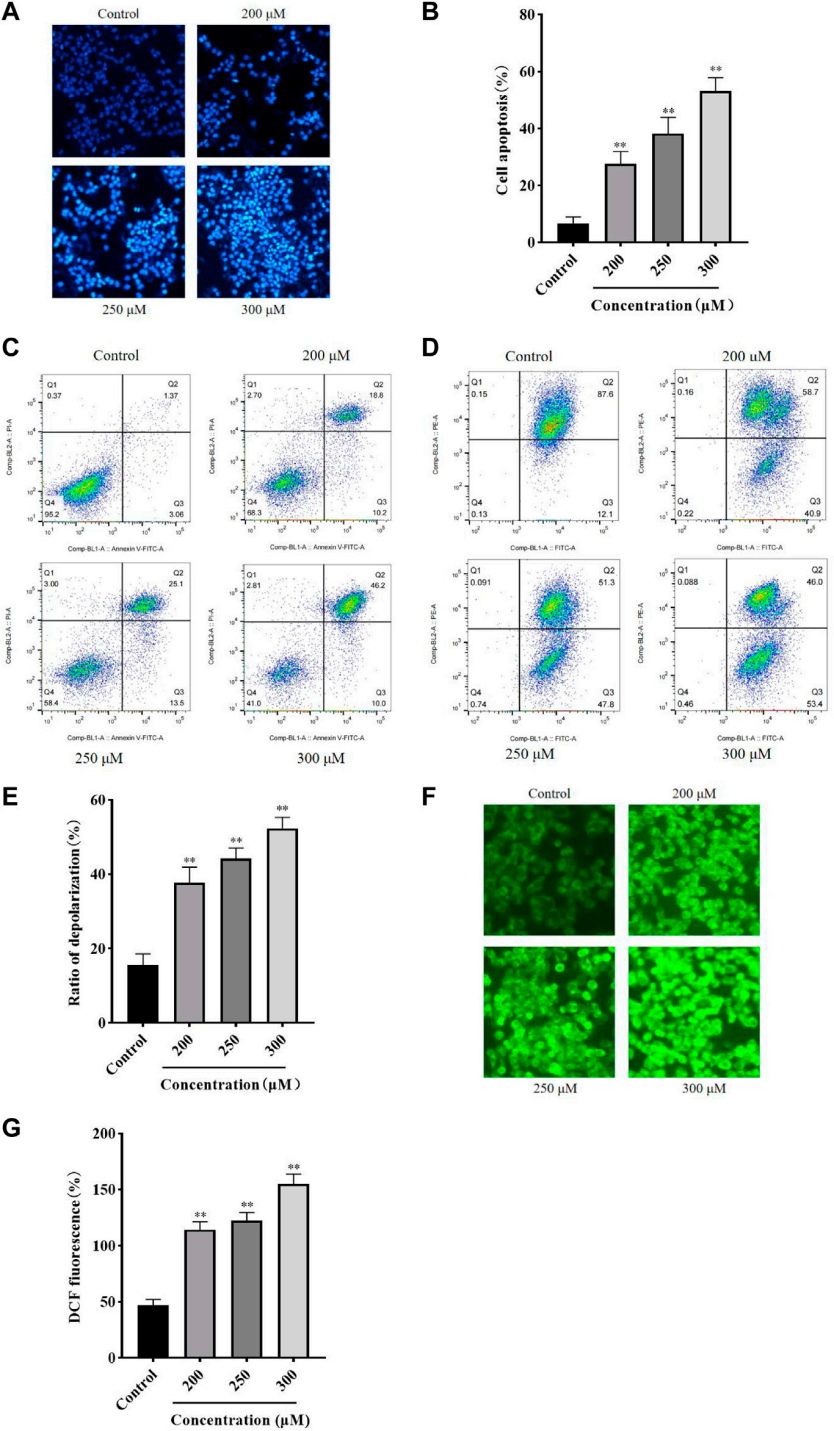
GA induced HCC1806 cells apoptosis accompanied by ROS accumulation and MMP depolarization. **(A–C)** Hoechst 33258 staining and flow cytometry were performed to assess the effect of GA on HCC1806 cells apoptosis. **(D,E)** The depolarization ratio of HCC1806 cells were detected by flow cytometry. **(F,G)** The ROS level of HCC1806 cells were assessed by a fluorescence microscope. Date were expressed as means ± SD. Compared with the control group, ^**^ *p* < 0.01.

Hoechst 33258 Staining and flow cytometry were performed to assess the effect of GA on HCC1806 cells apoptosis. As shown in Figure 2A, compared with the control group, cells in the GA intervention groups showed a strong fluorescence response, cytoplasmic condensation and nuclear incorporation, indicating an increased number of cells in an apoptotic state. Furthermore, as shown in Figures 2B,C, flow cytometry results indicated that GA induced HCC1806 cells apoptosis. Compared with the control group, the apoptotic cell rate of HCC1806 cells in GA treatment groups were significantly increased. The apoptotic rate in the control group was 4.43%, while in the GA treatment groups were 29%, 38.6% and 56.2%, respectively. In addition, the proportion of late apoptosis was higher than early apoptosis, these data indicated that GA-induced apoptosis was mainly achieved through late apoptosis.

### GA reduces intracellular MMP level

The JC-1 staining method was used to detect the cell MMP level. As shown in Figures 2D,E, the upper right quadrant represented the proportion of cells that did not depolarized, while the lower right quadrant represented the proportion of cells that depolarized. These results indicated that GA induced MMP depolarization. Compared with the control group, the MMP depolarization rate of HCC1806 cells in GA treatment groups were significantly increased. The MMP depolarization rate in the control group was 12.1%, while in the GA treatment groups were 40.9%, 47.8% and 53.4%, respectively. These results indicated that GA could decrease the MMP levels in HCC1806 cells and implied GA-induced apoptosis might be related to the mitochondrial apoptosis pathway.

### GA promotes intracellular ROS generation

The DCFH-DA fluorescent probe was used to detect intracellular ROS accumulation and assess cellular damage’s extent. As shown in Figures 2F,G, the green fluorescence of cells in GA treatment groups significantly increased compared with the control group. The levels of ROS in the control group was 46.94%, while in the GA treatment groups were 117.34%, 122.44%, and 154.72%, respectively. These results suggested that GA induced oxidative stress in HCC1806 cells.

### GA induces HCC1806 cells apoptosis *via* the mitochondrial pathway

**FIGURE 3:**
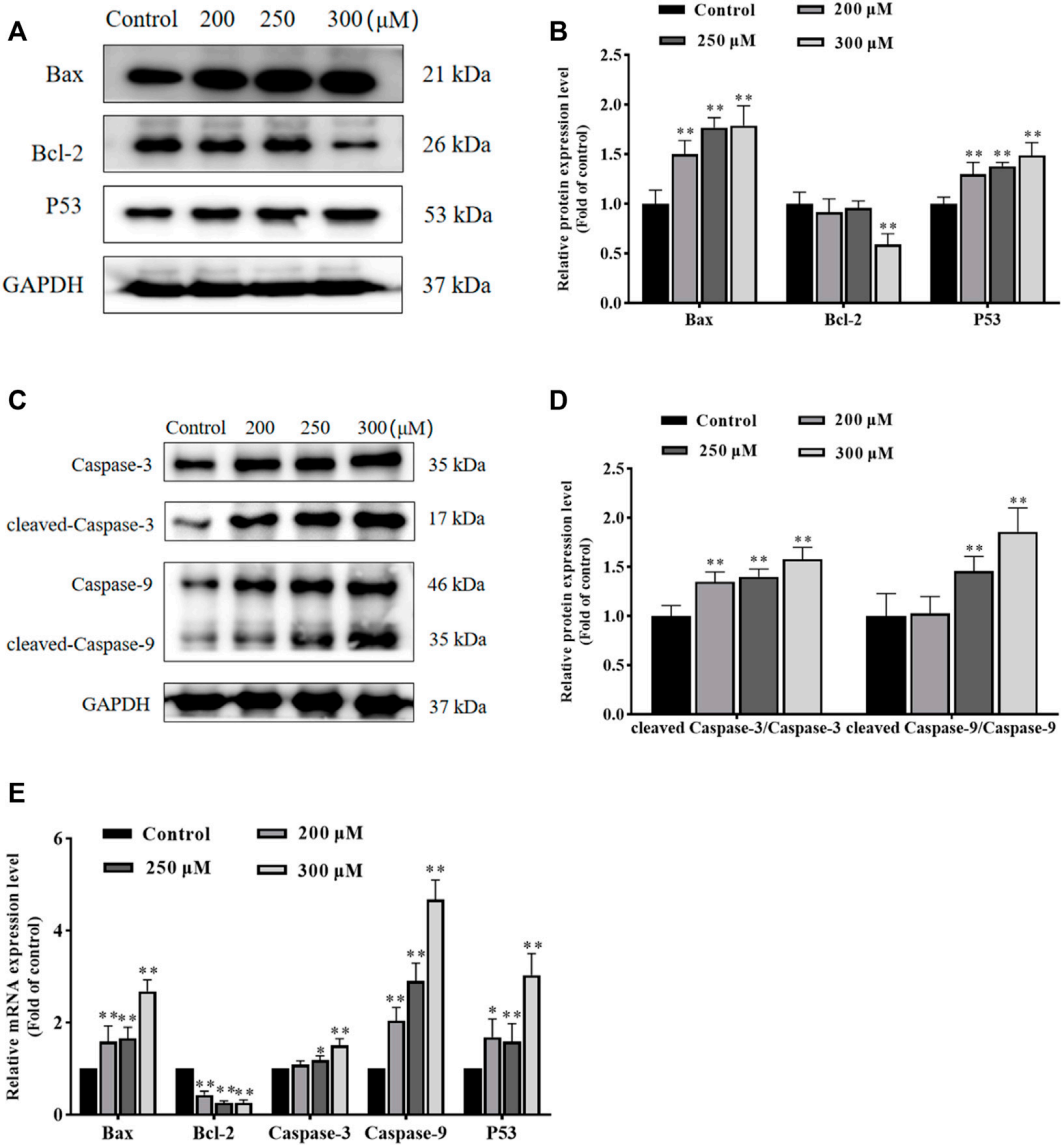
GA induces HCC1806 cells apoptosis *via* the mitochondrial pathway. **(A–D)** Effect of GA on the expression of Bax, Bcl-2, cleaved-Caspase-3, cleaved-Caspase-9 and P53 proteins in HCC1806 cells. **(E)** Effect of GA on the expression of Bax, Bcl-2, Caspase-3, Caspase-9 and P53 mRNA in HCC1806 cells. Date were expressed as means ± SD. Compared with the control group, ^*^ *p* < 0.05,^**^ *p* < 0.01.

To further investigate the effect of GA in promoting apoptosis and to assess whether this effect was associated with mitochondrial functional impairment, we examined the expression of related proteins and genes. As shown in Figures 3A–E, compared with the control group, the expression of Bax and P53 proteins in GA treatment groups were significantly increased, and the ratio of cleaved-Caspase-3/Caspase-3 and cleaved-Caspase-9/Caspase-9 were significantly increased, while the expression of Bcl-2 protein was significantly decreased. Meanwhile, compared with the control group, the expression of Bax, Caspase-3, Caspase-9 and P53 mRNA in GA treatment groups were significantly increased, while the expression of Bcl-2 mRNA was significantly decreased. These data indicated that GA induced HCC1806 cells apoptosis was related to the mitochondrial apoptosis pathway.

### GA induces HCC1806 cells apoptosis *via* the PI3K/AKT/EGFR and MAPK signaling pathways

**FIGURE 4:**
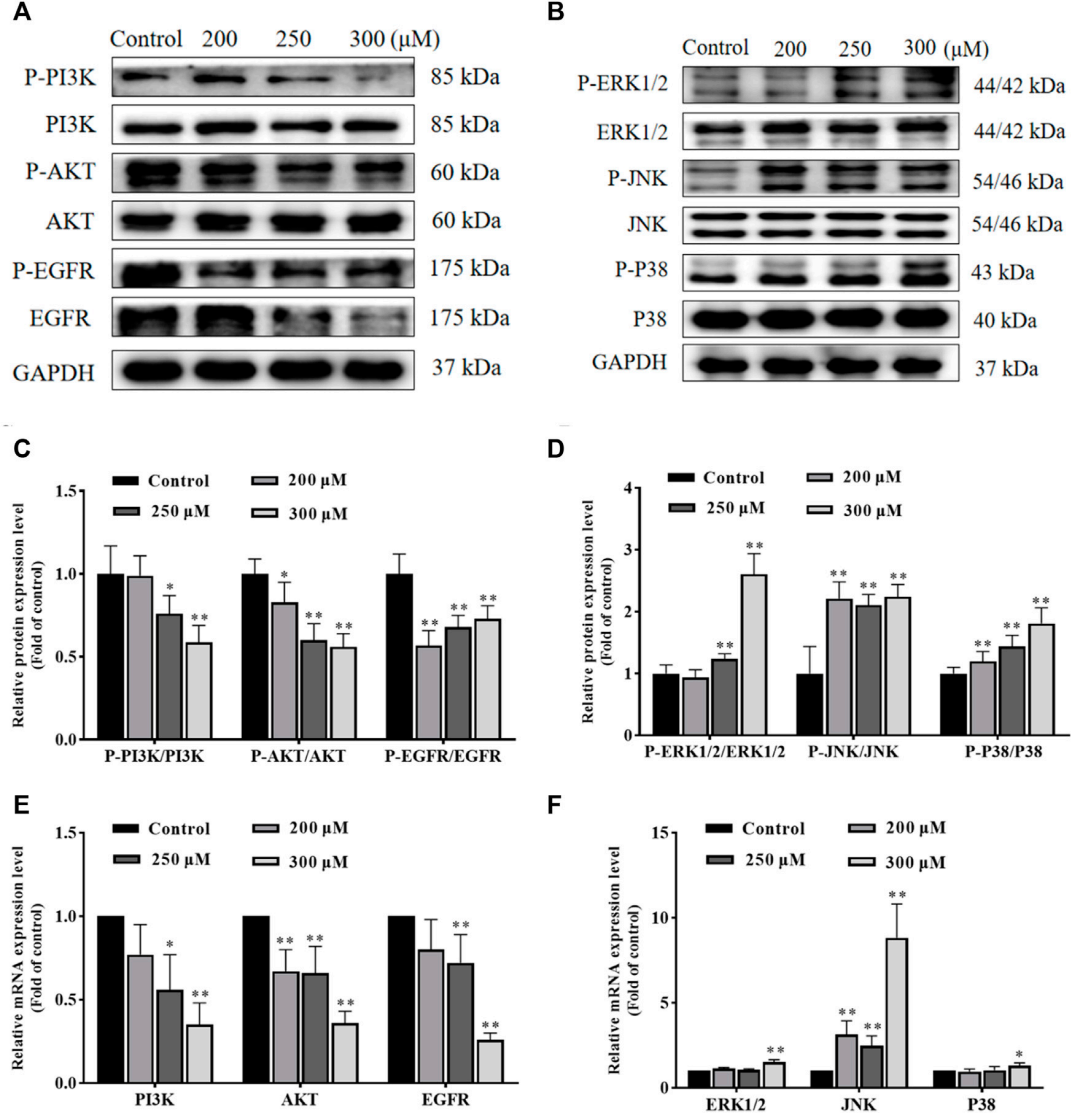
GA induces HCC1806 cells apoptosis *via* the PI3K/Akt/EGFR and MAPK signaling pathways. **(A–D)** Effect of GA on the expression of P-PI3K, P-AKT, P-EGFR, P-ERK1/2, P-JNK, and P-P38 proteins in HCC1806 cells. **(E,F)** Effect of GA on the expression of PI3K, AKT, EGFR, ERK1/2, JNK and P38 mRNA in HCC1806 cells. Date were expressed as means ± SD. Compared with the control group, ^*^ *p* < 0.05, ^**^ *p* < 0.01.

In order to investigate the possible molecular mechanisms of GA-induced apoptosis, we examined the expression of PI3K, P-PI3K, AKT, P-AKT, EGFR, P-EGFR, ERK1/2, P-ERK1/2, JNK, P-JNK, P38 and P-P38 proteins. As shown in Figures 4A–D, compared with the control group, the ratio of P-PI3K/PI3K, P-AKT/AKT and P-EGFR/EGFR in GA treatment groups were significantly decreased, while the ratio of P-ERK1/2/ERK1/2, P-JNK/JNK and P-P38/P38 in GA treatment groups were significantly increased. Furthermore, as shown in Figures 4E,F, the expression of PI3K, AKT and EGFR mRNA in GA treatment groups significantly decreased, while the expression of JNK, ERK1/2 and P38 mRNA in GA treatment groups significantly increased. These data suggested that GA induced HCC1806 cells apoptosis *via* modulating PI3K/AKT/EGFR and MAPK signaling pathways.

### Molecular docking showed high binding of GA with PI3K, AKT, EGFR, ERK1/2, JNK, and P38

**FIGURE 5:**
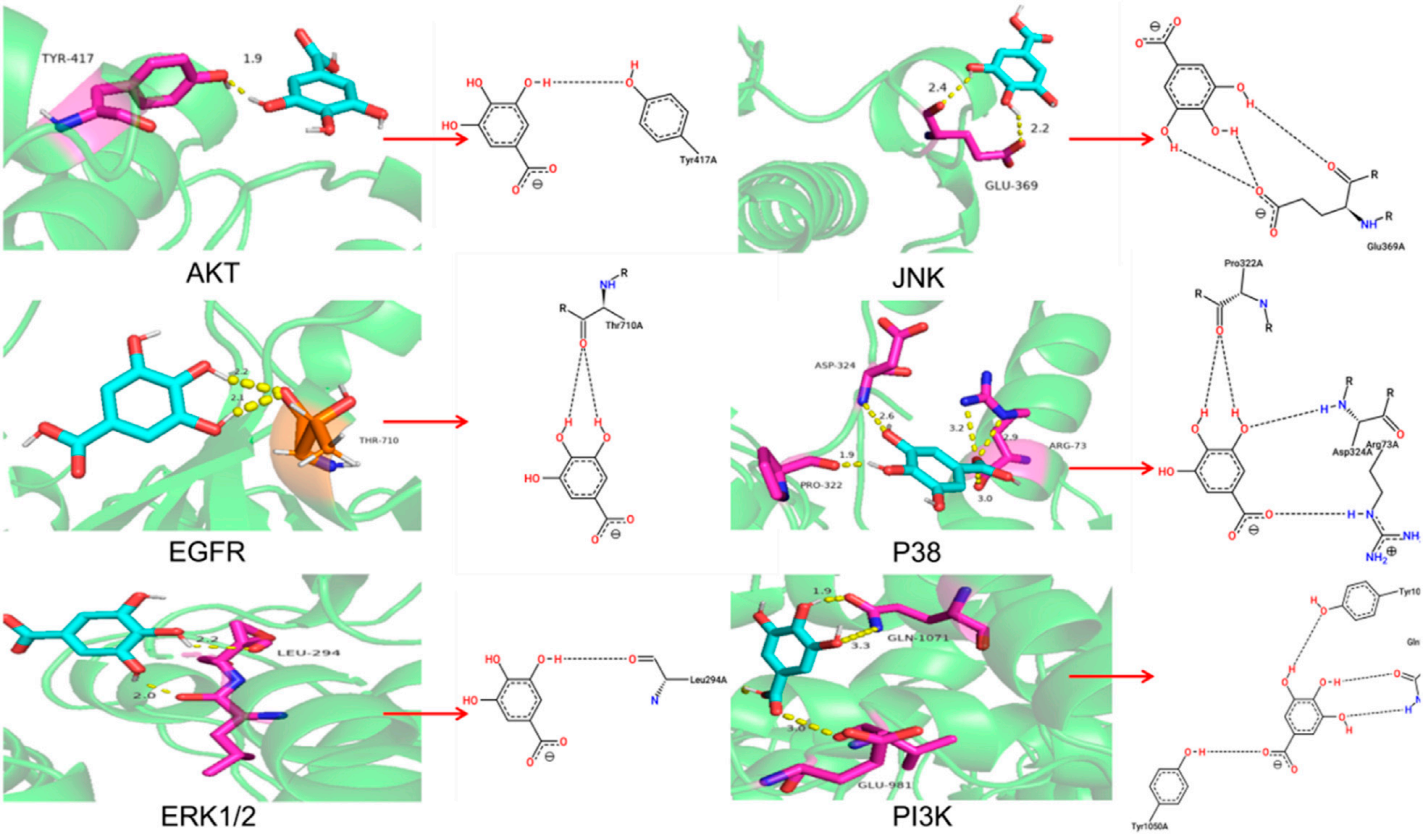
**(A)** Molecular docking diagram of GA and PI3K, AKT, EGFR, ERK1/2, JNK, and P38.

The molecular docking results were shown in Table 2 and Figure 5A. And the specific information of protein name, PDB ID, hydrogen bond (bond length) and docking binding energy were shown in Table 2. The binding energy of PI3K, AKT, EGFR, ERK1/2, JNK, and P38 are −7.32 kcal mol^−1^, −5.61 kcal mol^−1^, −4.64 kcal mol^−1^, −9.21 kcal mol^−1^, −4.39 kcal mol^−1^ and −7.63 kcal mol^−1^, respectively. These molecular docking results showed that GA and each target protein had good docking activity, which could stably dock in the active capsule of the receptor protein and form hydrogen bonds. Therefore, it could be suggested that GA has a high affinity for PI3K, AKT, EGFR, ERK1/2, JNK, and P38 from the docking studies.

**TABLE 2:** Information of molecular docking analysis.

Protein name	PDB ID	Hydrogen bond (bond length)	Docking binding energy/kcal·mol^−1^
PI3K	3APC	GLN-1071 (1.9, 3.3), GLU-981 (3.0)	−7.32
AKT	1GZK	TYR-417 (1.9)	−5.61
EGFR	3IKA	THR-710 (2.1, 2.2)	−4.64
ERK1/2	5LCJ	LEU-294 (2.0, 2.2)	−9.21
JNK	3V6R	GLU-369 (2.2, 2.4)	−4.39
P38	2GHL	ASP-324 (2.6), PRO-322 (1.9), ARG-73 (2.9, 3.0, 3.2)	−7.63

## Discussion

BC is one of the most prevalent malignancies in women worldwide and the leading cause of cancer deaths in women (Sung et al., 2021). TNBC is more prone to distant metastases and recurrence than other breast cancers, and the risk of recurrence is highest in the third year (Shah et al., 2012). Currently, surgery, chemotherapy and radiotherapy are commonly used to treat TNBC, but these methods have serious adverse effects and do not reduce the recurrence rate of TNBC (Kim et al., 2012; Beggs et al., 2014). Therefore, it is essential to find a safe and effective drug to treat TNBC. Previous studies have shown that GA can block the MDA-MB-231 cell cycle in the G_1_ phase, induce apoptosis through the P38/P21/P27 signaling axis, activate the ferroptosis pathway by inducing ROS production in MDA-MB-231 cells, and also promote cellular ROS generation and depolarization of mitochondrial membrane potential in combination with curcumin, thereby inducing MDA-MB-231 cells apoptosis (Lee et al., 2017; Moghtaderi et al., 2018; Khorsandi et al., 2020). In the present study, the effect of GA on the proliferation and apoptosis of HCC1806 cells was systematically investigated for the first time. We indicated that GA could inhibit viability and induce apoptosis of HCC1806 cells. Furthermore, it was also found that inhibition of the PI3K/AKT/EGFR signaling pathway and activation MAPK signaling pathway, which relied on the generation of ROS were involved in GA-induced apoptosis. To our knowledge, this is the first study to demonstrate that GA inhibits the viability and induces apoptosis of HCC1806 cells.

We first assessed the effect of GA on the proliferation of HCC1806 cells by MTT and plate clone formation assays. The results showed that GA could inhibit HCC1806 cells proliferation in a dose-and time-dependent manner (Zeng et al., 2020; Chen et al., 2021). Meanwhile, we found that the morphology of HCC1806 cells changed significantly after GA treatment, the number of HCC1806 cells decreased and the cells were in an apoptotic state. Furthermore, we found that GA blocked HCC1806 cells cycle in S phase. Therefore, we speculated that the inhibitory effect of GA on the proliferation of HCC1806 cells was related to the induction of apoptosis. Subsequently, we assessed the effect of GA on apoptosis of HCC1806 cells by Hoechst 33258 Staining and flow cytometry. Surprisingly, GA could induce apoptosis in HCC1806 cells. Therefore, the following experiments mainly explored the molecular mechanism of GA promoting apoptosis of HCC1806 cells.

Mitochondria plays a decisive role in cell apoptosis, and the mitochondrial apoptosis pathway is often accompanied by ROS accumulation and MMP depolarization (Song et al., 2016; Burke, 2017; Wen et al., 2019; Hengartner, 2020). We detected the ROS and MMP levels, and as we had expected, the ROS level significantly increased and the MMP significantly decreased after GA treatment. Bax is a pro-apoptotic protein that promotes apoptosis by disrupting mitochondrial membranes and creating permeable pores. Bcl-2 is an anti-apoptotic protein that inhibits apoptosis by maintaining the integrity of mitochondrial membranes. Both Bax and Bcl-2 work together to regulate cell apoptosis (Zhao et al., 2018; Estaphan et al., 2020). The Caspases family is a key target of the mitochondrial apoptosis pathway, Caspase-9 activated by mitochondria-associated proteins triggers a cascade of Caspases and further activates Caspase-3, ultimately leading to cell apoptosis (Hadisaputri et al., 2021). Accumulating evidence indicated that P53 plays a crucial role in cell apoptosis by regulating the expression of apoptosis-related proteins and activating the mitochondrial apoptosis pathway to induce apoptosis (Amaral et al., 2010; Kanapathipillai, 2018; Stein et al., 2019). Our data indicated that the ratio of cleaved-Caspase-3/Caspase-3, cleaved-Caspase-9/Caspase-9 were significantly increased, and the expression of Bax and P53 were also significantly increased, while the expression of Bcl-2 was significantly decreased after GA treatment. Moreover, the expression of Bax, P53, Caspase-3 and Caspase-9 mRNA were significantly increased, while the expression of Bcl-2 mRNA was significantly decreased after GA treatment. These results showed that GA promotes HCC1806 cells apoptosis is associated with the mitochondrial apoptosis pathway. And our research has proved that the apoptosis-promoting effect of GA on HCC1806 cells is related to mitochondrial apoptosis pathway for the first time.

PI3K/Akt signaling pathway is ubiquitous in a variety of organisms. It can regulate the cell cycle, participate in angiogenesis, promote the transition of epithelial cells to mesenchymal cells, influence chemoresistance and many other biological processes (Xu and Davis, 2017). This signaling pathway often over-express in a variety of malignancies such as breast, ovarian and lung cancers (Zhang et al., 2018; Guo et al., 2019; Zhang T et al., 2019). EGFR is a large molecular weight transmembrane glycoprotein with multiple structural regions that plays an essential role in tumor formation and growth, and it has became the critical target for targeted therapies in various tumors (Chong and Janne, 2013; Sabbah et al., 2020). MAPK signaling pathways, including ERK, JNK, and P38, regulate nearly all cellular functions and are frequently deregulated in various human cancers (Dhillon et al., 2007). Several types of research have proved that activation of ERK1/2 signaling could cause DNA damage and induce cell apoptosis (Cagnol and Chambard, 2010; Dubey et al., 2018). Abnormal P38MAPK function is associated with cancer development and patient survival time, it mediates the Fas/Fas L apoptosis pathway by promoting p53 and c-jun phosphorylation, which induces Bax translocation, thereby activating the mitochondrial apoptosis pathway and ultimately promoting apoptosis (Feng et al., 2018; Fei et al., 2020). JNK signaling pathway plays a key role in cell proliferation, apoptosis and tumor development, and activation of the JNK signaling pathway is an effective way to induce apoptosis and is closely linked to apoptosis (Xu and Davis, 2017; Zhou et al., 2021). Tumor cells are in a state of high ROS levels and show a higher sensitivity to changes in ROS, which could induce apoptosis when stimulated by internal and external factors to produce excessive ROS (Bekhet and Eid, 2015). Amounting evidence confirmed that excessive ROS accumulation can inhibit the PI3K/AKT signaling pathway and activate the MAPK signaling pathway, ultimately resulting in cell apoptosis. For example, Zhu et al. (2020) proved ROS accumulation could induce tumor cell apoptosis by inhibiting PI3K/Akt/mTOR signaling pathway. In addition, Isoliensinine was shown to induce apoptosis in TNBC MDA-MB-231 cells by increasing ROS generation and activating P38/JNK signaling pathway (Zhang et al., 2015). And Liu et al. (2015) demonstrated that palmitate induces H9c2 cell death by activating ROS/MAPK signaling pathway. Our data showed that the expression of P-PI3K, P-AKT, and P-EGFR proteins were significantly decreased, while the expression of P-ERK1/2, P-JNK, and P-P38 proteins were significantly increased after GA treatment. In addition, the expression of PI3K, AKT and EGFR mRNA were significantly decreased, the expression of JNK and P38 mRNA were significantly increased after GA treatment. Furthermore, molecular docking results indicated that GA highly affinity for PI3K, AKT, EGFR, ERK1/2, JNK, and P38. These results provided the first evidence that GA promotes HCC1806 cells apoptosis and is associated with the PI3K/AKT/EGFR and MAPK signaling pathways, and we found that ROS generation induced by GA is crucial for the inhibition of PI3K/AKT signaling pathway and activation of MAPK signaling pathway.

In conclusion, our study provided the first evidence that GA inhibited HCC1806 cells proliferation and induced HCC1806 cells apoptosis *via* the mitochondrial apoptosis pathway and induced ROS generation, which further inhibits PI3K/AKT/EGFR and activates MAPK signaling pathways. Hence, our study initially revealed the mechanism of GA-induced apoptosis in HCC1806 cells, and it is different from previous studies on the mechanism of GA action on TNBC cells, and it will provide some new references for GA to treat TNBC. Moreover, in combination with previous studies, we know that GA has a pro-apoptotic effect on many TNBC cells and can promote apoptosis through multiple signaling pathways, which suggests that GA is a promising drug for treating TNBC. However, the present study only preliminarily validated the effect of GA on HCC1806 cells *in vitro*. Therefore, the effects and the mechanisms of action *in vivo* (animals) need to be explored in further studies.

## Data Availability

The original contributions presented in the study are included in the article/supplementary material, further inquiries can be directed to the corresponding authors.
